# Occupational Stressors and Dual Health Burden: Associations Between Body Mass Index and Common Mental Disorders Among Hospital and Manufacturing Employees in Indonesia

**DOI:** 10.3390/ijerph23040495

**Published:** 2026-04-14

**Authors:** Herqutanto, Muchtaruddin Mansyur, Annisa Maulidina, Muhammad Abror Rizani Fahmi

**Affiliations:** 1 Department of Community Medicine, Faculty of Medicine, Universitas Indonesia, Jakarta Pusat 10310, Indonesia; muchtaruddin.mansyur@ui.ac.id (M.M.); abrorizani@gmail.com (M.A.R.F.); 2 Southeast Asian Ministers of Education Organization Regional Centre for Food and Nutrition (SEAMEO RECFON), Pusat Kajian Gizi Regional (PKGR) Universitas Indonesia, Jakarta Timur 13120, Indonesia; annisa.maulidina@seameo-recfon.org; 3 Mailman School of Public Health, Columbia University, New York City, NY 10032, USA

**Keywords:** occupational stressor, manufacture, hospital, body mass index, common mental disorder

## Abstract

**Highlights:**

**Public health relevance—How does this work relate to a public health issue?**

- Occupational stressors affect both physical and mental health across diverse work settings, and both impacts are a shared public health concern across occupational groups/industrial sectors. Since the COVID-19 pandemic, the need to observe the diversity of various occupational groups and their stressors has increased.
- Evidence comparing stress-related health outcomes across occupational groups is scarce—close to none.

**Public health significance—Why is this work of significance to public health?**

- Regardless of age, gender, type of company, and specific job roles, a higher severity of stress exposure in two distinctive occupational groups (hospital and manufacturing company workers) is significantly associated with an increase in their common mental disorder (CMD) scores.
- The relationship between occupational stressors and difference in workers’ BMI appears to be non-significant and context-dependent.

**Public health implications—What are the key implications or messages for practitioners, policy makers and/or researchers in public health?**

- Occupational health strategies should address psychosocial hazards alongside physical risks. Workplaces, regardless of the company type (in this study, hospital and manufacturing companies), should integrate a tailored approach rather than a blanket one-size-fits-all psychosocial management strategy into their existing health program.
- Mental health surveillance should be prioritized in occupational health policies.

**Abstract:**

This comparative cross-sectional study simultaneously investigated the dual health burden of body mass index (BMI) and common mental disorders (CMDs) driven by occupational stressors in two stepwise regression models. By classifying stress exposure into three clinically relevant tiers (low, moderate, and severe) in two distinctive populations—a hospital and a manufacturing company—we used the validated SDS-30 and SRQ-20 instruments. The robust multiple regression models uncovered a highly nuanced landscape of employee well-being that highlights the context-dependent nature of psychosocial hazards. The most compelling findings emerged from the interaction analyses, which demonstrated that the physical and mental consequences of severe stress do not impact the workforce uniformly. Regarding mental health, severe occupational stress proved to be a potent catalyst for CMD symptoms, but this psychological toll was significantly magnified within the hospital sector relative to the manufacturing environment. An opposite, yet equally context-dependent, pattern emerged regarding physical health. In the main-effects-adjusted model, the severity of occupational stressors did not demonstrate a statistically significant linear association with an overall increase in BMI. However, the interaction model revealed a hidden vulnerability: employees in operational field roles who report severe stress are highly susceptible to severe BMI increases compared with admin personnel. While administrative staff may face sedentary risks, field workers under severe stress likely endure higher physiological allostatic load, erratic shift patterns that disrupt circadian metabolic rhythms, and potentially poorer dietary coping mechanisms during active labor. This combination of physical exhaustion and severe psychological tension severely disrupts metabolic homeostasis, forcing the redistribution of adipose tissue and driving the observed BMI spike.

## 1. Introduction

Work-related stressors, namely any environmental trigger of stress in the workplace, negatively affect both employees and the company [1]. Defined as the adverse psychological and physiological reaction that occurs when job demands exceed an employee’s capabilities, resources, or coping mechanisms, occupational stress initiates complex biological cascades that dictate long-term health trajectories. Although the impact of stress from the workplace may vary depending on individual reactions, stress may induce the development of metabolic syndrome, which further promotes cardiovascular disease [2]. The chronic activation of the stress response system, particularly the hypothalamic–pituitary–adrenal (HPA) axis, results in the sustained release of glucocorticoids, most notably cortisol [3]. Elevated cortisol levels systematically disrupt metabolic homeostasis by promoting cellular insulin resistance, increasing the appetite for hypercaloric foods, and redistributing adipose tissue toward the visceral compartment [3]. Consequently, body mass index (BMI) serves as a critical, easily quantifiable proxy indicator of this metabolic disruption. Assessing BMI in the context of occupational stress allows for the early detection of pathways leading to metabolic syndrome and subsequent cardiovascular morbidity.

A significant effect of job strain, one out of many examples of work-related stressors, has been confirmed to be the risk of metabolic syndrome in workers [4]. Many studies [2,5,6,7] have confirmed the association between occupational stress and various components of metabolic syndrome, which refers to a cluster of central obesity (through BMI), hypertension, elevated triglycerides and low high-density lipoprotein (HDL) cholesterol, and hyperglycemia [7]. In a study performed in Norway [8], it was revealed that employees whose work was more sedentary were associated with metabolic syndrome and a higher risk of death from cardiovascular events. Additionally, a systematic review [9] confirmed that there is an association between occupational stress and the BMI of hospital employees as one specific occupational group. Furthermore, in a study on healthcare workers, more severe metabolic syndrome and cardiovascular disease risk factors were detected as the BMI of the employees increased [10].

There is a large amount of evidence emerging that focuses on the effects of various stressors on particular occupational groups, such as healthcare workers, petrochemical enterprise employees, and university staff members [2,6,9]. All this evidence highlights the importance of looking at incremental data offered by various occupational groups that differ in terms of the nature of their occupational physical activity to further observe stressors and metabolic risk factors. Unadjusted factors, such as job role types (for example, a cashier in an administrative role compared with a nurse in a hospital), might lead to confounding bias [2]. Therefore, further exploration of the health impacts of stressors in the workplace across different occupational populations is imperative.

Simultaneously, dysregulated stress responses directly contribute to the onset of common mental disorders (CMDs), a classification encompassing non-psychotic symptoms primarily characterized by anxiety and depression [11]. CMDs represent a growing global health crisis, fundamentally impairing an individual’s quality of life and resulting in profound economic losses due to absenteeism and diminished workplace productivity [2,12]. Despite the recognized severity of these conditions, existing studies in the occupational health literature frequently examine the impact of workplace stress on either metabolic indicators or mental health in strict isolation.

It is critical to recognize, however, that occupational stress is not inherently pathological. Rooted in the Yerkes–Dodson law, the relationship between stress arousal and human performance follows an inverted-U trajectory [13]. A complete absence of workplace stress can lead to “rust-out”, characterized by insufficient intellectual stimulation, reduced motivation, and cognitive disengagement [14]. Conversely, moderate stress exposure often manifests as “eustress”, which is a positive, mobilizing psychological state that enhances focus, resilience, and job satisfaction. Only when occupational stressors breach an individual’s coping capacity does the experience shift from eustress to “distress”, precipitating the metabolic and psychiatric deteriorations targeted in this study [15]. This nonlinear paradigm underscores the necessity of evaluating stress across a multitier continuum rather than a simple binary.

The occupational hazard profiles of different industries present unique challenges to employee well-being. Hospital employees, for example, face high levels of emotional labor, life-and-death decision-making responsibilities, and the circadian disruptions associated with irregular shift patterns [16]. Conversely, employees in the manufacturing sector contend with persistent physical hazards, rigid quantitative production targets, and environmental stressors such as noise and chemical exposures [17]. There remains a conspicuous scarcity of comparative epidemiological evidence examining both physical and psychological burdens simultaneously across distinctly different industrial sectors. Given these highly divergent hazard profiles, it is imperative to investigate the multi-faceted dynamics of health outcomes triggered by workplace stressors to determine if specific occupational contexts exacerbate the damage caused by psychological tension.

The primary objective of this comparative cross-sectional study is to identify the precise associations between occupational stressor levels and the dual burden of BMI alterations and CMD symptoms across two distinct working populations: hospital and manufacturing employees. By classifying stress exposure into three standardized severity tiers (low, moderate, severe) and using two different numerical outcomes (BMI and CMD scores), this study seeks to overcome the methodological limitations of oversimplification secondary to BMI classification and the dichotomous outcome of CMD presence; instead, we aim to establish clear dose–response gradients. We have two hypotheses: First, we hypothesize that higher tiers of occupational stress are positively and significantly associated with an increase in BMI and an escalation in the severity of CMD symptoms, independent of confounding demographic variables such as age and gender. Second, we hypothesize that the strength and magnitude of the association between occupational stress and these dual health outcomes are significantly modified by the type of workplace (in our study, hospital vs. manufacturing company) and the employee’s specific job classification (administrative vs. field-related roles), indicating that occupational health trajectories are fundamentally context-dependent.

## 2. Methodology

### 2.1. Participants

Our comparative, cross-sectional study involved employees from two distinct organizational environments: a tertiary care hospital and a large-scale manufacturing facility located in Indonesia. We recruited participants from only one hospital and one manufacturing company. Data were systematically collected during the mandatory Annual Medical Check-Up (MCU) administered in 2024 for the hospital cohort and 2024 for the manufacturing plant cohort. The implementation of this annual MCU is an obligatory legal requirement mandated by the Indonesian Minister of Manpower and Transmigration Regulation No. PER.02/MEN/1980 [18]. Because the MCU is legally enforced by organizational management and the state, the risk of selection bias typical of voluntary survey research is heavily mitigated, ensuring exceptionally high participation rates with randomization.

A total population sampling approach was utilized, targeting all eligible employees scheduled for the MCU during the respective study periods. Employees were excluded only if they were absent during the entire period in which the MCU took place. A total of 733 employees provided their written informed consent in be involved in the study and completed all the required clinical and psychological assessments, yielding a final analytical sample of 392 hospital employees and 341 manufacturing employees. Post hoc power analyses indicate that a sample size of 733 provides sufficient statistical power (exceeding 80%) to detect small-to-moderate effect sizes in multiple linear regression frameworks, assuming an alpha level of 0.05.

### 2.2. Instrument and Measurement

During the clinical assessment phase of the MCU, physical anthropometry was measured directly by certified nurses using calibrated digital scales and wall-mounted stadiometers to measure height and total body weight to calculate the employees’ body mass index (BMI). This direct measurement protocol strictly eliminates the risk of self-report bias, which frequently compromises the validity of BMI data in large-scale occupational surveys. Body mass index (BMI) was calculated continuously as weight in kilograms divided by the square of height in meters (kg/m^2^), and it serves as one out of the two outcome variables.

For the purpose of this study, the employees from both groups, from one hospital situated in Jakarta and a manufacturing company situated in Jakarta, were invited to complete two different questionnaires at their respective MCU site. The questionnaires assessing mental health and stressor exposure were self-administered via a secure digital platform. The participants accessed the instruments through a QR code provided at the MCU registry desk and completed them on mobile devices prior to concluding their clinical examinations. The primary exposure variable, occupational stressor, was assessed using the Stress Diagnosis Survey (SDS-30), a robust 30-item instrument formally adopted under the Indonesian Ministerial Regulation No. 5 of 2018 for the assessment of workplace psychosocial hazards. The instrument evaluates six distinct domains of occupational stress: role ambiguity, role conflict, quantitative workload, qualitative workload, career development, and responsibility for others. To ensure adequate analytical distribution and to capture potential dose–response gradients, the continuous total scores were aggregated and categorized into three tiers based on established psychometric scoring guidelines: low (score < 10), moderate (score 10–24), and severe (score ≥ 25). Another instrument, the World Health Organization’s Self-Reporting Questionnaire (SRQ-20), was also utilized during the MCU. The SRQ-20 consists of 20 dichotomous (yes/no) questions designed to screen for non-psychotic psychiatric disturbances, specifically targeting symptoms of depression, anxiety, and somatic distress in low–middle-income countries (LMICs), including Indonesia. The aggregate SRQ-20 score (range 0–20) was utilized as a continuous outcome variable (CMD score), which preserves statistical variance and allows for the precise linear modeling of mental health symptom severity. Detailed questions of the SDS-30 and SRQ-20 questionnaires are available in the Supplementary Materials.

This study was approved by the Ethics Committee of the Faculty of Medicine, University of Indonesia.

### 2.3. Data Analysis

All data transformations, descriptive statistics, and inferential modeling were conducted using RStudio (Posit Software, PBC, Version 2026.01.0+392). Descriptive statistics were generated to summarize the sociodemographic profiles across the three stressor levels. Differences between groups were assessed using analysis of variance (ANOVA) for continuous variables and Pearson’s Chi-squared tests for categorical variables.

Multiple linear regression models were constructed in a stepwise, hierarchical manner to evaluate the primary hypotheses for both BMI and CMD score outcomes. Crude models (Model 1) were initially fitted to assess the unadjusted association between the 3-level stressor and the outcomes. Adjusted models (Model 2) were subsequently constructed, controlling for potential confounding by age, gender, workplace, and job classification. To account for potential heterogeneity across work environments, interaction terms were included between occupational sectors (e.g., manufacturing vs. hospital) and stress levels. This enabled the model to test for non-uniform slopes, ensuring that sector-specific vulnerabilities were not masked by a simple main-effects assumption. The interaction models (Model 3) were developed by introducing mathematical interaction terms between the stressor level and the potential modifiers (Stressor level × Workplace; Stressor level × Job Classification) to formally test for effect modification. To note, age and gender are strictly confounders throughout the entire analysis, while workplace and job classification begin as simple confounders in Model 2 and are subsequently tested as effect modifiers in Model 3.

The overall explanatory power of the models was assessed using the adjusted coefficient of determination (adjusted R^2^). Statistical significance was established a priori at an alpha level of 0.05, and 95% confidence intervals were reported for all unstandardized beta coefficients (β). Age and gender were included in the analysis model as confounding factors.

## 3. Results

### 3.1. Sociodemographic Details and Characteristics of the Study Participants

Table 1 outlines the baseline characteristics of the 731 participating employees, stratified by the three-tier occupational stressor classification. The population ultimately fell into the low (n = 252, 34.5%), moderate (n = 453, 62.0%), or severe (n = 26, 3.6%) stress categories. Given the small number of participants that fell within the “severe stressor” group compared with those in the “low” and “moderate” groups, further interpretation of the association between stressor level and the outcome of interest (both BMI and CMD score) should proceed with this acknowledgement. The mean age of the cohort was 38 years (SD = 9), with the severe stress group exhibiting a significantly higher mean age of 41 years (*p* = 0.021). The gender distribution was relatively even (53% female, 47% male) and did not differ significantly across stress levels (*p* = 0.12). A striking divergence emerged regarding the source of extreme stress: employees in the manufacturing sector constituted the vast majority of the severe stress cohort (20 out of 26 individuals, or 77%), whereas hospital employees dominated the low-stress baseline (*p* < 0.001). Unadjusted initial comparisons indicated that both BMI and CMD scores worsened significantly as the reported stressor level increased (*p* = 0.039 and *p* < 0.001, respectively).

### 3.2. Employees’ Stressor Levels and Body Mass Index (BMI)

Linear regression evaluated the impact of occupational stressor level on BMI (Table 2). In the crude analysis (Model 1), using the low stressor group as a reference, individuals in the severe stress group demonstrated a statistically significant absolute increase in mean BMI (β = 2.4, 95% CI: 0.31 to 4.4). However, upon adjusting for covariates including age, gender, workplace, and job classification (Model 2), occupational stressor severity no longer maintained a statistically significant main effect on BMI. The magnitude of the severe stressor coefficient dropped from 2.4 to 2.0, and the confidence interval crossed the null threshold (95% CI: −0.13 to 4.1). Age remained a significant positive predictor of BMI (β = 0.05, *p* = 0.011).

The addition of interaction terms (Model 3) uncovered critical effect modification masked in the main-effects model. While the interaction between the level of stressor sourced in the workplace and the manufacturing sector was not significant, the interaction between stressor level and job classification revealed an obscure vulnerability, though it should be carefully interpreted given the wide confidence intervals and the disproportionate small number of individuals in the “severe” stressor group. The interaction term for severe stress × field classification yielded a statistically significant beta coefficient of 6.4 (95% CI: 1.6 to 11.0; *p* = 0.031). This indicates that while severe stressor does not uniformly increase BMI across the entire workforce, employees working in operational field roles experience a profound, disproportionate escalation in body mass when exposed to severe occupational stress compared with their administrative counterparts. Statistical significance was set at a *p*-value < 0.05. Despite the non-significant findings for BMI, the final model was deemed the most rigorous specification for defending the observed results as a true reflection of the study population.

**Figure 1:**
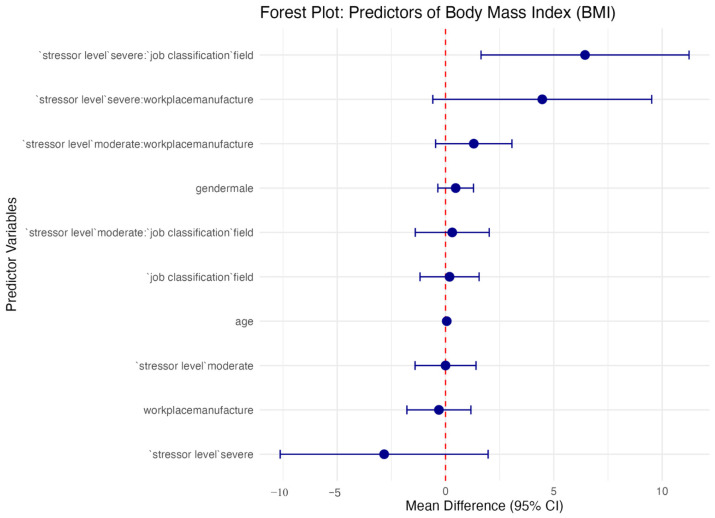
Adjusted multiple linear regression with interaction terms estimating Body Mass Index (BMI).

Figure 1 shows the adjusted multiple linear regression with interaction terms to estimate BMI. The overall explanatory power of the final interaction model for BMI was modest (R^2^ = 0.036, adjusted R^2^ = 0.023). If we compared it with the adjusted R^2^ in the main-effects model (adjusted R^2^ = 0.010), this slight increase (0.010 to 0.023) in the added variance may be explained by the interaction terms. This indicates that while the model identifies specific occupational vulnerabilities, such as the interactive effect between severe stressors and field work as their job classification, the variables included in this analysis account for approximately 2.3% of the total variance in employees’ body mass index. To explain the relatively low explanatory power of this regression model, it is necessary to consider the impact of unmeasured confounding factors not captured in this occupational dataset, such as individual dietary intake, work and sleep patterns, and out-of-work physical activity.

### 3.3. Stressor Level of Employees and Common Mental Disorders (CMDs)

Conversely, the severity level of occupational stressor proved to be a robust, highly significant predictor of CMD symptoms across all model specifications (Table 3). In the crude model, relative to the low stressor baseline, the moderate group exhibited elevated CMD scores (β = 0.99, 95% CI: 0.49 to 1.5), while the severe group demonstrated sharp symptom escalation (β = 2.5, 95% CI: 1.2 to 3.9). After comprehensive adjustment for sociodemographic covariates (Model 2), the dose–response relationship remained intact and highly significant (*p* < 0.001). The adjusted mean difference in SRQ-20 scores was 1.0 point higher for the moderate group and 2.7 points higher for the severe group. Crucially, the interaction analysis (Model 3) exposed significant effect modification by workplace context (*p* < 0.001). The model isolated the main effect of severe stressor (which, in the presence of the interaction term, represents the effect specifically within the reference hospital sector) at a striking β = 7.2 (95% CI: 4.2 to 10.0). However, the interaction term for severe × manufacture produced a strong negative coefficient (β = −5.9, 95% CI: −9.1 to −2.7). This mathematical relationship reveals that while severe stressor drives an escalation in common mental disorder symptoms among hospital personnel, this acute psychological deterioration is significantly blunted among manufacturing employees. However, related to the small numbers of participants in the “severe” stressor group (Table 1), the interpretation should be made with caution.

**Figure 2:**
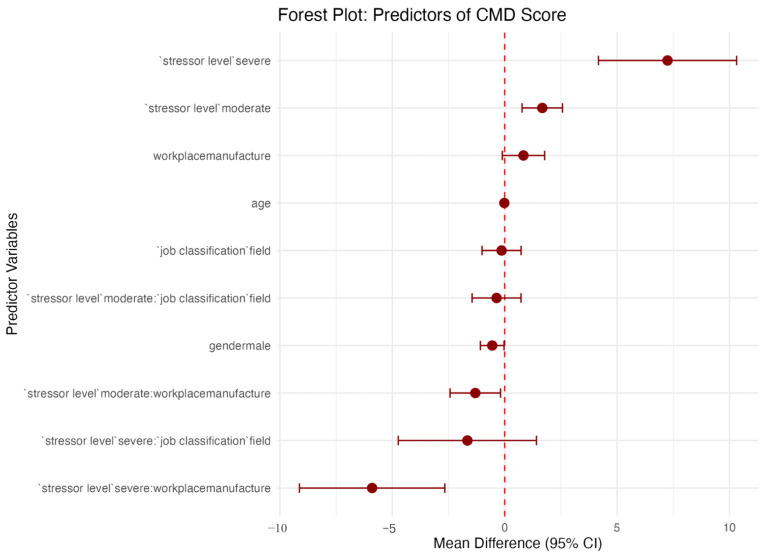
Adjusted multiple linear regression with interaction terms estimating CMD score.

Figure 2 shows the adjusted multiple linear regression with interaction terms in estimating CMD score. The fully specified interaction model accounted for a higher proportion of variance in mental health outcomes (CMD scores) compared with the physical health model (BMI), yielding an adjusted R^2^ of 0.047. There is a slight increase from the main-effects model compared with the final interaction model (adjusted R^2^ = 0.032 to adjusted R^2^ = 0.047), also explained by the interaction terms. The adjusted R^2^ demonstrates that the modeled occupational factors and their interactions explain 4.7% of the variance in the reported CMD scores (resulted from submitted SRQ-20 questionnaire) across the study population.

## 4. Discussion

This study evaluated the two health indicators, physical (BMI) and psychological (CMD), associated with occupational stressors in two distinct industrial environments. The findings indicate a divergent relationship: while occupational stressor is strongly linked to common (non-psychosis) mental disorder symptoms in this cohort, its association with BMI is notably more conditional and appears limited to specific subgroups. While these associations provide important insights into employees’ mental health and well-being, they should be interpreted within the context of the study’s cross-sectional design, which describes only a snapshot of health at a single point in time rather than a definitive causal pathway.

Contrary to the initial assumption that the high emotional labor and life-and-death responsibilities of tertiary hospital environments would drive the highest levels of distress [19], our results demonstrate that employees in the manufacturing sector were disproportionately represented in the severe stressor group (77% vs. 23%; *p*-value < 0.001). This phenomenon can be attributed to the fundamental difference in the nature of “industrial” versus “service” stressors. While hospital stressors are often qualitative and interpersonal, according to the SDS-30 instrument, rooted in role ambiguity and emotional regulation, manufacturing stressors in the Indonesian context are primarily quantitative and structural. Manufacturing environments are uniquely characterized by rigid production targets and “human–machine” interactions that enforce a relentless work pace, often leaving employees with minimal autonomy over their tasks [20]. Furthermore, the physical environment of manufacturing plants involves chronic exposure to noise, vibration, and extreme temperatures, which function as persistent physiological stressors that compound psychological tension [20]. This suggests that while the volume of stressors may be higher in manufacturing companies, the psychological resilience or institutional support mechanisms may differ across sectors. These findings highlight the potential for sector-specific mental health strategies, although further longitudinal research is required to confirm whether these stressors directly lead to clinical psychological disorders over time.

The disproportionate severe stressor in manufacturing may also reflect the “payment–reward imbalance” often found in industrial labor sectors. Unlike healthcare professionals who may experience higher social prestige and intrinsic reward from patient outcomes, manufacturing workers often engage in repetitive, monotonous labor with worse job security and limited opportunities for career development [17]. This is consistent with recent findings in Southeast Asian industrial hubs, where escalating production demands to meet global market requirements have significantly increased the subjective workload and fatigue of industrial laborers compared with service-sector cohorts [21]. Consequently, while healthcare stress is undoubtedly pervasive, the structural and environmental constraints of the manufacturing sector appear more likely to push employees beyond their coping capacity and into the “severe distress” threshold [22].

Regarding physical health outcomes, the main-effects model did not yield a statistically significant linear association between occupational stressor severity and an overall increase in BMI. The absence of a uniform BMI escalation across the stress continuum contradicts prior studies that assumed a strictly linear dose–response [9]. This discrepancy can be explained by integrating the Yerkes–Dodson theoretical framework. For the majority of the cohort (62%) reporting moderate stressors sourced from their workplace, these demands likely operate as “eustress”, which refers to a mobilizing force that promotes adaptive coping without triggering chronic HPA axis dysregulation. Consequently, systemic metabolic disruption does not universally occur until stress breaches the “severe” distress threshold [23].

Even then, the interaction models demonstrate that this physiological deterioration is not generalized; it is highly concentrated among operational field workers. As shown in Model 3 (Table 2), the interaction term for severe stress * field classification yielded a statistically significant coefficient (β = 6.4, *p*-value = 0.031). Field personnel experiencing severe psychological distress likely suffer from compounding allostatic load, where physical exertion, erratic shift patterns that disrupt circadian metabolic rhythms, and stress-induced hypercaloric dietary coping mechanisms synergistically drive rapid adipose tissue accumulation [24]. Administrative staff, despite facing sedentary risks, do not exhibit this acute vulnerability to stress-induced weight gain. Given the wide confidence intervals, in addition to the non-statistically significant association between severe stressors and the BMI model, the final model that includes interaction terms should be interpreted cautiously.

Regarding mental health outcomes, occupational stress functioned as a robust catalyst for CMD symptoms across all models. However, the trajectory of this psychological deterioration was profoundly context-dependent. The Model 3 interaction analysis (Table 3) isolated a striking main effect of severe stress (β = 7.2) within the reference hospital sector, contrasted against a significant negative interaction term for manufacturing employees (β = −5.9). This reveals that severe stress precipitates a significant escalation in CMD symptoms, specifically among hospital personnel. This acute psychiatric vulnerability is likely driven by the unique psychosocial hazards intrinsic to clinical settings, including high emotional labor, moral distress, and the gravity of life-and-death patient care responsibilities [25]. When stress breaches a severe threshold in a hospital setting, the interpersonal and high-stakes nature of the work translates rapidly into acute anxiety and depressive symptomology. In contrast, the structural rigidity of the manufacturing sector may channel distress through alternative pathways, such as physical fatigue or musculoskeletal complaints, rather than immediate psychiatric disturbances [20].

By classifying stress exposure into three clinically relevant tiers (low, moderate, and severe) using the validated SDS-30 instrument in both environments, the multiple regression models illuminated a highly nuanced landscape of employee well-being that emphasizes the context-dependent nature of psychosocial hazards. While the regression models had isolated significant covariates, particularly in identifying the association between severe occupational stressor and the employees’ CMD scores, the overall proportion of variance explained by these R2 models remains relatively low. The final BMI and CMD interaction models yielded adjusted R2 values of 0.023 and 0.047, respectively. The primary objective of this analysis was to estimate the isolated contribution of workplace stressors’ severity level on health outcomes, rather than to construct a comprehensive predictive model of an employee’s total health trajectory. It is critical to place the limited explanatory power within its proper epidemiological context in this study; that is, the current findings reflect specific associative pathways rather than assumptive and predictive relationships.

Given the fact that both BMI and CMD are highly multifactorial in terms of their endpoints, interpretation from the results of this study should consider the unobserved covariates not yet captured in this occupational dataset, including daily caloric intake and physical activity outside working hours, non-occupational factors related to psychiatric history, and genetic predisposition. The omission of these robust, off-the-clock determinants inherently limits the maximum achievable R2, precluding the models from generating strong individualized predictions. Crucially, a constrained R2 does not negate the methodological validity or the public health relevance of the detected associations. The robust beta coefficients and narrow confidence intervals in the CMD model confirm a reliable, dose–response gradient between occupational stress and psychological deterioration. This validates a distinct association wherein elevated workplace stress corresponds predictably to worse mental health outcomes, even though occupational stress represents only a singular fraction of the broader etiological matrix determining overall employee well-being.

### Strengths and Limitations

What believe that our study is pioneering in the field as we simultaneously assess the association between stressor level and both BMI (physical outcome) and common mental disorders (psychological outcome). We use a specific methodological approach (linear regression) to identify two outcomes using one exposure of interest, the stressor level, in two distinctive occupational groups. The stressor level identified in this study used the standardized SDS-30 questionnaire for stressor severity assessment in both hospital and manufacturing companies, making up the strength of this study among other research studies that are still limited to investigating just one occupational group in silo. In addition to our ANOVA and stepwise multiple linear regression analysis, we utilized modifier interactions, specifically workplace difference (hospital vs. manufacturing company) and stratified employee job roles (administrative vs. field-related job roles), to make our model more robust and unique.

However, the findings of this study must be interpreted within the context of several methodological limitations. First, the cross-sectional design fundamentally precludes the establishment of strict temporal causality. It is impossible to definitively determine whether severe workplace stress precipitated the onset of CMD symptoms, or if individuals already suffering from early-stage CMDs perceive their workplace stressors more acutely, reflecting an element of reverse causality.

Second, the regression models evaluating BMI and CMD yielded relatively low adjusted R^2^ values. In the disciplines of public health and occupational epidemiology, human adiposity and mental health are immensely complex, multifactorial phenotypes. While chronic occupational stress theoretically upregulates cortisol and promotes weight gain, actual BMI outcomes are fundamentally mediated by a vast array of lifestyle factors not captured during a standard medical check-up, including detailed caloric intake, leisure-time physical activity, exact sleep duration, and substance use. The absence of these variables contributes directly to residual confounding and the modest explanatory power of the models. However, the relatively narrow confidence intervals surrounding the baseline (“low stressor” group) and intermediate stressor group estimates indicate that the models possess sufficient precision to evaluate the independent associations of the included variables, even if they cannot holistically predict an individual’s exact BMI or CMD score.

Finally, regarding generalizability, while this study encompasses two distinct and economically vital industrial sectors within Indonesia, the cultural nuances of workplace hierarchy, stigma regarding mental health reporting, and labor protections in Southeast Asia may differ significantly from Western occupational cohorts. Consequently, caution must be exercised before extrapolating these specific interaction effects to vastly different socioeconomic regions.

## 5. Conclusions

In this study, we identify a significant association between occupational stressor and common mental disorder (CMD) symptoms among Indonesian employees in two distinctive occupational groups. Our findings suggest that mental health is highly sensitive to workplace stress; however, the interaction analysis in our model uncovered a sector-specific paradox. Manufacturing employees reported severe stressors in their workplace more frequently than hospital employees, yet the magnitude of the association with CMD symptoms was more pronounced among hospital workers.

The association between stress and BMI appears to be highly context-dependent, manifesting primarily through specific interactions such as those observed in field-based roles. Methodologically, the use of a three-level stressor categorization (“low”, “moderate”, and “severe”) and interaction modeling provided a more nuanced understanding of these relationships than that achieved with previous binary models. Nevertheless, the relatively low explanatory power of the models and the cross-sectional nature of the data necessitate a cautious interpretation of our findings. These findings report the identification of critical links and potential vulnerabilities within the workforce; they do not establish definitive causal trajectories. The performance of longitudinal studies in the future is essential to determine the temporal direction of these associations and explore the role of unmeasured variables, such as diet, lifestyle, and sleep patterns, in the broader etiological matrix of employee health.

## Figures and Tables

**Table 1:** Sociodemographic details and characteristics of the study participants.

Characteristic	Overall n = 731 ^1^	Low n = 252 ^1^	Moderate n = 453 ^1^	Severe n = 26 ^1^	*p*-Value ^2^
Age (Years)	38 (9)	38 (10)	37 (9)	41 (9)	0.021
Gender					0.12
female	386 (53%)	146 (58%)	228 (50%)	12 (46%)	
male	345 (47%)	106 (42%)	225 (50%)	14 (54%)	
Workplace Sector					<0.001
hospital	392 (54%)	169 (67%)	217 (48%)	6 (23%)	
manufacture	339 (46%)	83 (33%)	236 (52%)	20 (77%)	
Job Classification					0.2
admin	436 (60%)	143 (57%)	274 (60%)	19 (73%)	
field	295 (40%)	109 (43%)	179 (40%)	7 (27%)	
Body Mass Index (kg/m^2^)	26.6 (5.1)	26.1 (5.1)	26.8 (5.0)	28.5 (6.2)	0.039
CMD Symptoms (SRQ-20 Score)	2.24 (3.33)	1.54 (3.07)	2.53 (3.32)	4.08 (4.52)	<0.001

^1^ Mean (SD); n (%); ^2^ one-way analysis of means; Pearson’s Chi-squared test.

**Table 2:** Multiple linear regression models estimating the association between occupational stressor levels and body mass index (BMI). The table presents unstandardized beta coefficients (β), 95% confidence intervals (CIs), and *p*-values across three-tiered model specifications. The crude model assesses the unadjusted main effect of the primary stressor exposure. The adjusted model controls for baseline confounding by including age, gender, workplace sector, and job classification as covariates. The interaction model further incorporates two-way mathematical interaction terms (Stressor Level × Workplace Sector; Stressor Level × Job Classification) to test for context-dependent effect modification.

	Crude Model			Adjusted Model			Interaction Model		
Characteristic	β	95% CI	*p*-Value	β	95% CI	*p*-Value	β	95% CI	*p*-Value
Occupational Stressor Level			0.039			0.10			0.5
low	—	—		—	—		—	—	
moderate	0.69	−0.10, 1.5		0.62	−0.18, 1.4		0.00	−1.4, 1.4	
severe	2.4	0.31, 4.4		2.0	−0.13, 4.1		−2.8	−7.6, 2.0	
Age (Years)				0.05	0.01, 0.10	0.011	0.06	0.01, 0.10	0.008
Gender						0.4			0.3
female				—	—		—	—	
male				0.35	−0.47, 1.2		0.47	−0.35, 1.3	
Workplace Sector						0.11			0.7
hospital				—	—		—	—	
manufacture				0.71	−0.16, 1.6		−0.31	−1.8, 1.2	
Job Classification						0.2			0.8
admin				—	—		—	—	
field				0.56	−0.27, 1.4		0.19	−1.2, 1.6	
Stressor Level × Workplace Sector									0.12
moderate × manufacture							1.3	−0.46, 3.1	
severe × manufacture							4.5	−0.59, 9.5	
Stressor Level × Job Classification									0.031
moderate × field							0.31	−1.4, 2.0	
severe × field							6.4	1.6, 11	

Abbreviation: CI = confidence interval.

**Table 3:** Multiple linear regression models estimating the association between occupational stressor levels and common mental disorder (CMD) symptoms, measured continuously via the SRQ-20 score. The table details unstandardized beta coefficients (β), 95% confidence intervals (CIs), and *p*-values for three distinct models. The crude model evaluates the unadjusted association between stress severity and CMD scores. The adjusted model accounts for potential residual confounding by adjusting for age, gender, workplace sector, and job classification. The interaction model formally tests for effect modification by introducing interaction terms between the stressor exposure and organizational contexts (Stressor Level × Workplace Sector; Stressor Level × Job Classification).

	Crude Model			Adjusted Model			Interaction Model		
Characteristic	β	95% CI	*p*-Value	β	95% CI	*p*-Value	β	95% CI	*p*-Value
Occupational Stressor Level			<0.001			<0.001			<0.001
low	—	—		—	—		—	—	
moderate	0.99	0.49, 1.5		1.0	0.51, 1.5		1.7	0.77, 2.6	
severe	2.5	1.2, 3.9		2.7	1.3, 4.0		7.2	4.2, 10	
Age (Years)				−0.02	−0.04, 0.01	0.2	−0.02	−0.04, 0.01	0.2
Gender						0.076			0.038
female				—	—		—	—	
male				−0.48	−1.0, 0.05		−0.56	−1.1, −0.03	
Workplace Sector						0.5			0.082
hospital				—	—		—	—	
manufacture				−0.20	−0.76, 0.36		0.83	−0.11, 1.8	
Job Classification						0.14			0.8
admin				—	—		—	—	
field				−0.40	−0.93, 0.13		−0.14	−1.0, 0.73	
Stressor Level × Workplace Sector									<0.001
moderate × manufacture							−1.3	−2.4, −0.19	
severe × manufacture							−5.9	−9.1, −2.7	
Stressor Level × Job Classification									0.5
moderate × field							−0.36	−1.5, 0.73	
severe × field							−1.7	−4.7, 1.4	

Abbreviation: CI = confidence interval.

## Data Availability

The data presented in this study are available from the corresponding author on request. The raw individual-level data are not publicly available due to institutional data privacy and medical record data protection policy.

## Supplementary Materials

The following supporting information can be downloaded at https://www.mdpi.com/article/10.3390/ijerph23040495/s1.
