# Supplementary material for: Occupational Stressors and Dual Health Burden: Associations Between Body Mass Index and Common Mental Disorders Among Hospital and Manufacturing Employees in Indonesia
Source: Int J Environ Res Public Health. 2026 Apr 14;23(4):495. doi: 10.3390/ijerph23040495 (PMC13115938; doi:10.3390/ijerph23040495)
Supplement: Supplementary file 1 [file ijerph-23-00495-s001.zip › SDS-30 Questionnaire (Bahasa Indonesia) - Google Form.pdf]

SDS-30

Kuisioner ini dirancang untuk mengetahui
sejauh mana kondisi hidup yang sifatnya pribadi menjadi sumber stres Anda.
Untuk setiap pertanyaan Anda harus memilih seberapa sering kondisi yang dimaksud tersebut menjadi sumber stres.

Petunjuk Pengisian
Nilai setiap pernyataan menggunakan **skala 1 sampai 7**, di mana angka yang lebih tinggi menunjukkan tingkat stres yang lebih besar:
1 = Sangat tidak setuju (Bila kondisi yang diuraikan **tidak pernah** menimbulkan stres)
7 = Sangat setuju (Bila kondisi yang diuraikan **selalu** menimbulkan stres)

Waktu yang dibutuhkan untuk pengisian kurang lebih 5-8 menit.

* Menunjukkan pertanyaan yang wajib diisi

1. Nama Lengkap *

2. Usia (dalam tahun) *

3. Tanggal Lahir (bulan, hari, tahun): *

   Contoh: 7 Januari 2019

4. Jenis Kelamin *

   Tandai satu oval saja.

   - Laki-laki
   - Perempuan

5. Masa Kerja (dalam tahun) *

6. Jabatan/Posisi Pekerjaan *

7. Wilayah Kerja *

8. Status Pekerjaan (Petrochina/lainnya)

9. Pendidikan Terakhir *

10. Status Pernikahan (Single/Menikah/Duda/Janda) *

11. 1. Tujuan tugas-tugas dan pekerjaan saya tidak jelas: *

Tandai satu oval saja.

	1	2	3	4	5	6	7	
Bila	◯	◯	◯	◯	◯	◯	◯	Bila kondisi yang diuraikan selalu menimbulkan stres

12. 2. Saya mengerjakan tugas-tugas atau proyek-proyek yang tidak perlu *

Tandai satu oval saja.

	1	2	3	4	5	6	7	
Bila	◯	◯	◯	◯	◯	◯	◯	Bila kondisi yang diuraikan selalu menimbulkan stres

13. 3. Saya harus membawa pulang pekerjaan ke rumah setiap sore hari atau akhir pekan agar dapat mengejar waktu *

Tandai satu oval saja.

	1	2	3	4	5	6	7	
Bila	◯	◯	◯	◯	◯	◯	◯	Bila kondisi yang diuraikan selalu menimbulkan stres

14. 4. Tuntutan-tuntutan mengenai mutu pekerjaan terhadap saya keterlaluan *

Tandai satu oval saja.

	1	2	3	4	5	6	7	
Bila	◯	◯	◯	◯	◯	◯	◯	Bila kondisi yang diuraikan selalu menimbulkan stres

15. 5. Saya tidak mempunyai kesempatan yang memadai untuk maju dalam organisasi ini *

Tandai satu oval saja.

	1	2	3	4	5	6	7	
Bila	◯	◯	◯	◯	◯	◯	◯	Bila kondisi yang diuraikan selalu menimbulkan stres

16. 6. Saya bertanggung jawab untuk pengembangan karyawan lain *

Tandai satu oval saja.

	1	2	3	4	5	6	7	
Bila	◯	◯	◯	◯	◯	◯	◯	Bila kondisi yang diuraikan selalu menimbulkan stres

17. 7. Saya tidak jelas kepada siapa harus melapor dan/atau siapa yang melapor kepada saya *

Tandai satu oval saja.

	1	2	3	4	5	6	7	
Bila	◯	◯	◯	◯	◯	◯	◯	Bila kondisi yang diuraikan selalu menimbulkan stres

18. 8. Saya terjepit ditengah-tengah antara atasan dan bawahan saya *

Tandai satu oval saja.

	1	2	3	4	5	6	7	
Bila	◯	◯	◯	◯	◯	◯	◯	Bila kondisi yang diuraikan selalu menimbulkan stres

19. 9. Saya menghabiskan waktu terlalu banyak untuk pertemuan-pertemuan yang tidak penting yang menyita waktu saya *

Tandai satu oval saja.

	1	2	3	4	5	6	7	
Bila	◯	◯	◯	◯	◯	◯	◯	Bila kondisi yang diuraikan selalu menimbulkan stres

20. 10. Tugas-tugas yang diberikan kepada saya kadang-kadang terlalu sulit dan/atau terlalu kompleks *

Tandai satu oval saja.

	1	2	3	4	5	6	7	
Bila	◯	◯	◯	◯	◯	◯	◯	Bila kondisi yang diuraikan selalu menimbulkan stres

21. 11. Kalau saya ingin naik pangkat, saya harus mencari pekerjaan pada satuan kerja lain *

Tandai satu oval saja.

	1	2	3	4	5	6	7	
Bila	◯	◯	◯	◯	◯	◯	◯	Bila kondisi yang diuraikan selalu menimbulkan stres

22. 12. Saya bertanggung jawab untuk membimbing dan/atau membantu bawahan saya menyelesaikan problemnya *

Tandai satu oval saja.

	1	2	3	4	5	6	7	
Bila	◯	◯	◯	◯	◯	◯	◯	Bila kondisi yang diuraikan selalu menimbulkan stres

23. 13. Saya tidak mempunyai wewenang untuk melaksanakan tanggung jawab pekerjaan saya *

Tandai satu oval saja.

	1	2	3	4	5	6	7	
Bila	◯	◯	◯	◯	◯	◯	◯	Bila kondisi yang diuraikan selalu menimbulkan stres

24. 14. Jalur perintah yang formal tidak dipatuhi *

Tandai satu oval saja.

	1	2	3	4	5	6	7	
Bila	◯	◯	◯	◯	◯	◯	◯	Bila kondisi yang diuraikan selalu menimbulkan stres

25. 15. Saya bertanggung jawab atas semua proyek pekerjaan dalam waktu bersamaan yang hampir tidak dapat dikendalikan *

Tandai satu oval saja.

	1	2	3	4	5	6	7	
Bila	◯	◯	◯	◯	◯	◯	◯	Bila kondisi yang diuraikan selalu menimbulkan stres

26. 16. Tugas-tugas tampaknya makin hari menjadi semakin kompleks *

Tandai satu oval saja.

	1	2	3	4	5	6	7	
Bila	◯	◯	◯	◯	◯	◯	◯	Bila kondisi yang diuraikan selalu menimbulkan stres

27. 17. Saya merugikan kemajuan karir saya dengan menetap pada organisasi ini *

Tandai satu oval saja.

	1	2	3	4	5	6	7	
Bila	◯	◯	◯	◯	◯	◯	◯	Bila kondisi yang diuraikan selalu menimbulkan stres

28. 18. Saya bertindak atau membuat keputusan-keputusan yang mempengaruhi keselamatan dan kesejahteraan orang lain *

Tandai satu oval saja.

	1	2	3	4	5	6	7	
Bila	◯	◯	◯	◯	◯	◯	◯	Bila kondisi yang diuraikan selalu menimbulkan stres

29. 19. Saya tidak mengerti sepenuhnya apa yang diharapkan dari saya *

Tandai satu oval saja.

	1	2	3	4	5	6	7	
Bila	◯	◯	◯	◯	◯	◯	◯	Bila kondisi yang diuraikan selalu menimbulkan stres

30. 20. Saya melakukan pekerjaan yang diterima oleh satu orang tapi tidak diterima oleh yang lain *

Tandai satu oval saja.

	1	2	3	4	5	6	7	
Bila	◯	◯	◯	◯	◯	◯	◯	Bila kondisi yang diuraikan selalu menimbulkan stres

31. 21. Saya benar-benar mempunyai pekerjaan yang lebih banyak daripada yang biasanya dapat dikerjakan dalam sehari *

Tandai satu oval saja.

	1	2	3	4	5	6	7	
Bila	◯	◯	◯	◯	◯	◯	◯	Bila kondisi yang diuraikan selalu menimbulkan stres

32. 22. Organisasi mengharapkan saya melebihi keterampilan dan/atau kemampuan yang saya miliki *

Tandai satu oval saja.

	1	2	3	4	5	6	7	
Bila	◯	◯	◯	◯	◯	◯	◯	Bila kondisi yang diuraikan selalu menimbulkan stres

33. 23. Saya hanya mempunyai sedikit kesempatan untuk berkembang dan belajar pengetahuan dan keterampilan baru dalam pekerjaan saya *

Tandai satu oval saja.

	1	2	3	4	5	6	7	
Bila	◯	◯	◯	◯	◯	◯	◯	Bila kondisi yang diuraikan selalu menimbulkan stres

34. 23. Saya hanya mempunyai sedikit kesempatan untuk berkembang dan belajar pengetahuan dan keterampilan baru dalam pekerjaan saya *

Tandai satu oval saja.

	1	2	3	4	5	6	7	
Bila	◯	◯	◯	◯	◯	◯	◯	Bila kondisi yang diuraikan selalu menimbulkan stres

35. 24. Tanggung jawab saya dalam organisasi ini lebih mengenai orang daripada barang *

Tandai satu oval saja.

	1	2	3	4	5	6	7	
Bila	◯	◯	◯	◯	◯	◯	◯	Bila kondisi yang diuraikan selalu menimbulkan stres

36. 25. Saya tidak mengerti bagian yang diperankan pekerjaan saya dalam memenuhi tujuan organisasi keseluruhan *

Tandai satu oval saja.

	1	2	3	4	5	6	7	
Bila	◯	◯	◯	◯	◯	◯	◯	Bila kondisi yang diuraikan selalu menimbulkan stres

37. 26. Saya menerima permintaan-permintaan yang saling bertentangan dari satu orang atau lebih *

Tandai satu oval saja.

	1	2	3	4	5	6	7	
Bila	◯	◯	◯	◯	◯	◯	◯	Bila kondisi yang diuraikan selalu menimbulkan stres

38. 27. Saya merasa bahwa saya betul-betul tidak punya waktu untuk istirahat berkala *

Tandai satu oval saja.

	1	2	3	4	5	6	7	
Bila	◯	◯	◯	◯	◯	◯	◯	Bila kondisi yang diuraikan selalu menimbulkan stres

39. 28. Saya kurang terlatih dan/atau kurang pengalaman untuk melaksanakan tugas-tugas saya secara memadai *

Tandai satu oval saja.

	1	2	3	4	5	6	7	
Bila	◯	◯	◯	◯	◯	◯	◯	Bila kondisi yang diuraikan selalu menimbulkan stres

40. 29. Saya merasa mandeg dalam karir saya *

Tandai satu oval saja.

	1	2	3	4	5	6	7	
Bila	◯	◯	◯	◯	◯	◯	◯	Bila kondisi yang diuraikan selalu menimbulkan stres

41. 30. Saya bertanggung jawab atas hari depan (karir) orang lain *

Tandai satu oval saja.

	1	2	3	4	5	6	7	
Bila	◯	◯	◯	◯	◯	◯	◯	Bila kondisi yang diuraikan selalu menimbulkan stres

Konten ini tidak dibuat atau didukung oleh Google.

Google Formulir
