# Supplementary material for: Occupational Stressors and Dual Health Burden: Associations Between Body Mass Index and Common Mental Disorders Among Hospital and Manufacturing Employees in Indonesia
Source: Int J Environ Res Public Health. 2026 Apr 14;23(4):495. doi: 10.3390/ijerph23040495 (PMC13115938; doi:10.3390/ijerph23040495)
Supplement: Supplementary file 1 [file ijerph-23-00495-s001.zip › SRQ-20 Questionnaire (Bahasa Indonesia) - Google Form.pdf]

SRQ-20

Petunjuk Pengisian Kuesioner ***(Kerahasian Isi Dijamin)***

1. Pada halaman ini terdapat 20 pernyataan. Dengan menggunakan penyataan-penyataan di *bawah kami ingin mendapatkan kesan mengenai keadaan Anda secara umum selama* ***4 minggu terakhir***

2. Beri tanda/pilihlah jawaban dari nilai yang paling sesuai bagi Anda

Waktu yang dibutuhkan untuk pengisian kurang lebih 5 menit.

* Menunjukkan pertanyaan yang wajib diisi

1. Nama Lengkap *

2. Usia (dalam tahun) *

3. Tanggal Lahir (bulan, hari, tahun): *

Contoh: 7 Januari 2019

4. Jenis Kelamin *

Tandai satu oval saja.

- Laki-laki
- Perempuan

5. Masa Kerja (dalam tahun) *

6. Jabatan/Posisi Pekerjaan *

7. Wilayah Kerja *

8. Status Pekerjaan (Petrochina/lainnya)

9. Pendidikan Terakhir *

10. Status Pernikahan (Single/Menikah/Duda/Janda) *

11. 1. Apakah anda **sering mengalami sakit kepala** *

Tandai satu oval saja.

- Ya
- Tidak

12. 2. Apakah anda merasa **tidak nafsu makan**? *

Tandai satu oval saja.

- Ya
- Tidak

13. 3. Apakah tidur anda **tidak nyenyak**? *

Tandai satu oval saja.

- Ya
- Tidak

14. 4. Apakah anda mudah **merasa ketakutan** ? *

Tandai satu oval saja.

- Ya
- Tidak

15. 5. Apakah tangan anda gemetaran ? *

Tandai satu oval saja.

- Ya
- Tidak

16. 6. Apakah anda merasa **gugup, ada tekanan dan ketakutan** ? *

Tandai satu oval saja.

- Ya
- Tidak

17. 7. Apakah **pencernaan anda buruk** ? *

Tandai satu oval saja.

- Ya
- Tidak

18. 8. Apakah anda merasa **sulit untuk berkonsentrasi** ? *

Tandai satu oval saja.

- Ya
- Tidak

19. 9. Apakah anda merasa **tidak bahagia** ? *

Tandai satu oval saja.

- Ya
- Tidak

20. 10. Apakah anda lebih sering **menangis daripada biasanya** ? *

Tandai satu oval saja.

- Ya
- Tidak

21. 11. Apakah anda merasa **kesulitan untuk menikmati aktifitas harian** anda ? *

Tandai satu oval saja.

- Ya
- Tidak

22. 12. Apakah anda merasa **sulit untuk membuat keputusan** ? *

Tandai satu oval saja.

- Ya
- Tidak

23. 13. Apakah pekerjaan rutin anda membuat anda **menderita** ? *

Tandai satu oval saja.

- Ya
- Tidak

24. 14. Apakah anda merasa **tidak bisa berperan secara positif** dalam kehidupan anda ? *

Tandai satu oval saja.

- Ya
- Tidak

25. 15. Apakah anda **kehilangan minat** atas sesuatu ? *

Tandai satu oval saja.

- Ya
- Tidak

26. 16. Apakah anda merasa diri anda sebagai **orang yang tidak berguna** ? *

Tandai satu oval saja.

- Ya
- Tidak

27. 17. Apakah pernah terlintas dalam benak anda keinginan untuk **mengakhiri hidup** ? *

Tandai satu oval saja.

- Ya
- Tidak

28. 18. Apakah anda merasa **kelelahan sepanjang waktu** ? *

Tandai satu oval saja.

- Ya
- Tidak

29. 19. Apakah anda merasa **tidak nyaman dengan lambung** anda ? *

Tandai satu oval saja.

- Ya
- Tidak

30. 20. Apakah anda **mudah merasa lelah** ? *

Tandai satu oval saja.

- Ya
- Tidak

Konten ini tidak dibuat atau didukung oleh Google.

Google Formulir
